# The immune modifying effects of amino acids on gut-associated lymphoid tissue

**DOI:** 10.1186/2049-1891-4-27

**Published:** 2013-07-30

**Authors:** Megan R Ruth, Catherine J Field

**Affiliations:** 1 Department of Agricultural, Food and Nutritional Science, 4-126A Li Ka Shing Health Research Innovation Centre, University of Alberta, Edmonton, AB T6G 2E1, Canada

**Keywords:** Amino acids, Arginine, Epithelium, Glutamate, Glutamine, Gut-associated lymphoid tissue, Intestine, Mucosa

## Abstract

The intestine and the gut-associated lymphoid tissue (GALT) are essential components of whole body immune defense, protecting the body from foreign antigens and pathogens, while allowing tolerance to commensal bacteria and dietary antigens. The requirement for protein to support the immune system is well established. Less is known regarding the immune modifying properties of individual amino acids, particularly on the GALT. Both oral and parenteral feeding studies have established convincing evidence that not only the total protein intake, but the availability of specific dietary amino acids (in particular glutamine, glutamate, and arginine, and perhaps methionine, cysteine and threonine) are essential to optimizing the immune functions of the intestine and the proximal resident immune cells. These amino acids each have unique properties that include, maintaining the integrity, growth and function of the intestine, as well as normalizing inflammatory cytokine secretion and improving T-lymphocyte numbers, specific T cell functions, and the secretion of IgA by lamina propria cells. Our understanding of this area has come from studies that have supplemented single amino acids to a mixed protein diet and measuring the effect on specific immune parameters. Future studies should be designed using amino acid mixtures that target a number of specific functions of GALT in order to optimize immune function in domestic animals and humans during critical periods of development and various disease states.

## Introduction

It is well established that protein deficiency suppresses the immune response and increases susceptibility to infection. In fact, protein energy malnutrition is hypothesized to be the leading contributor to immune deficiency globally [1]. Although the requirement for protein to support immunity is well defined and part of current recommendations, only recently have investigators begun to explore the potential use of individual dietary amino acids to optimize immune function. Early evidence suggested that amino acids are important energy substrates for immune cells [2-5] and for antioxidant defense mechanisms [6]. There are also critical health states (i.e. burns, trauma, infection, total parenteral (TPN) feeding) or periods of development (i.e. weaning, pregnancy) where it is now accepted that some dietary non-essential amino acids become conditionally essential. These include arginine, glutamine, glutamate, glycine, proline, taurine and cysteine [7]. This change in need for these amino acids in the diet may be due in part because of their effects on immune function.

The intestine serves not only as the main site of nutrient absorption and amino acid metabolism, but is also the largest immune organ in the body. The intestinal epithelium, while facilitating nutrient absorption, also has a major role in protecting the host from oral pathogens, inducing oral tolerance and maintaining a healthy interaction with commensal bacteria. Indeed both protein and single amino acid deficiencies have been shown to impair the physical integrity and growth of the intestinal epithelium, as well as alter the immune response [8]. This manuscript will review our current understanding of Gut- Associated Lymphoid Tissue (GALT) and examine the immunomodulatory effects of specific amino acids on immunity that occurs or originates in the intestine.

### The intestinal barrier and the gut associated immune system

**Figure 1:**
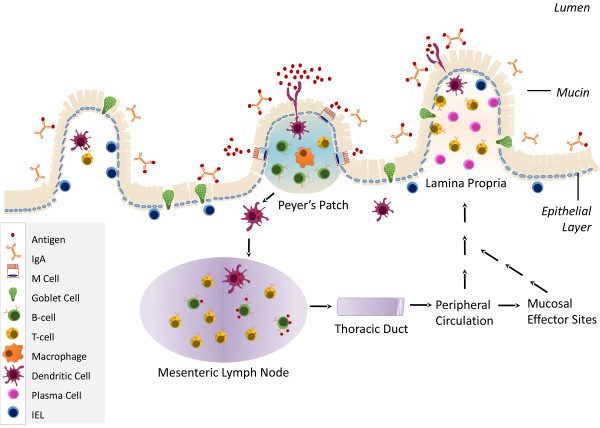
Diagram of the Gut-Associated Lymphoid Tissue.

GALT, the largest immune organ in the body of humans and domestic animals, contains a variety of immune cell types from the innate and acquired immune systems (as reviewed by [9]). Because of the proximity to the microbiome and the immediate contact with food, it is continually exposed to both ‘normal’ and potentially dangerous antigens. Accordingly, GALT develops in a manner that allows non-pathogenic substances, such as commensal bacteria, to survive and enables tolerance to food antigens, while protecting the host from pathogenic organisms and other potentially toxic substances [9]. GALT is considered a component of the mucosal immune system and is composed of aggregated tissue including Peyer’s patches (PPs) and solitary lymphoid follicles, and non-aggregated cells in the lamina propria, intestinal epithelial cells (IECs), intraepithelial lymphocytes (IELs), as well as mesenteric lymph nodes (MLNs) [9]. Collectively, GALT plays a critical role in the development of the systemic immune response. As a primary site of antigen exposure it primes naïve T- and B-lymphocytes that develop into effector cells which migrate from the intestine to other sites of the body to protect against immune challenges, such as invading pathogens (Figure 1).

GALT has an important role in first line mucosal defenses. The epithelium is protected from large pathogens or particles by a layer of mucin, a glycoprotein secreted from the specialized goblet cell within the endothelium [10]. The IELs are dispersed among the IECs that line intestinal villi and both cell types play a role in gut immune function (Figure 1). Tight junction proteins, such as claudin, occludin and ZO-1, determine the mucosal permeability and regulate the flow of solutes between the IECs [10]. IECs are involved in the intestinal immune response and some consider them an integral part of GALT. They can activate or suppress IELs via secretion of anti-microbial peptides, cytokines and chemokines or through the processing and presentation of antigen in the context of MHC Class I and MHC class II molecules to the IELS [11]. IELs are primarily T-cells but have functions distinct from peripheral T-cells [12]. The types of T-cells present vary widely by species and disease states [13], but the majority are CD8+, CD45RO + (antigen mature), and express adhesion molecules that are thought to be homing signals [12]. In mice and cows/calves, but not humans, the majority of T-cells are γδ T-cell Receptor + (TCR+) and the remainder are αβTCR + [13-15]. The primary role of γδTCR + cells is to induce tolerance and the primary role of the αβTCR + cells is to induce IgA production [13]. The difference between species may be related to the degree of exposure to the microbiota and different dietary exposure and requirements.

The PPs are lymphoid aggregates that line the intestine and colon and are the primary inductive sites of the mucosal humoral immune response (Figure 1) [16]. The follicle associated epithelium (FAE) layer of the PP contains highly specialized cells called microfold or M cells that continually sample the intestinal contents bringing them in contact with the resident immune cells (primarily B-cells and small numbers of macrophages, dendritic cells and T-cells) [17]. Dendritic cells can also extend through the intestinal epithelial cells to directly sample antigen [18]. Antigen presenting cells, particularly dendritic cells, migrate from the PP or epithelium to the MLN where they educate naïve T-cells [19]. The MLNs act as the interphase between the peripheral immune system and the gut and it is believed that they are the primary sites of oral tolerance induction [17]. Oral tolerance is mechanistically defined as the process by which dendritic cells present peptides to CD4+ T-cells and through a series of signals (cell surface and secreted) induce regulatory T-cells and subsequently the tolerance to the antigen/peptide. In rats, MLN are composed primarily of T-helper cells (55%), but also contain cytotoxic T-cells (15%), B-cells (25%) and dendritic cells (5%) [20]. Pigs have slightly different phenotypes, with approximately 12% CD4+ CD8+, 25-28% CD4+ (single positive), 27-32% CD8+ (single positive) [21] and the rest composed of B-cells and other antigen presenting cells [22].

After exposure to antigen in the PPs and MLNs, immune cells circulate in the periphery and migrate to other mucosal effector sites and home back to the lamina propria (Figure 1) [23]. This is the major effector component of GALT as these cells are antigen mature and primed to respond to foreign antigens. The lamina propria is comprised primarily of IgA secreting plasma cells and effector T-cells (50% T-helper and 30% cytotoxic T-cells) [24]. Secretory IgA (sIgA) is the most abundant immunoglobulin in the mammalian intestine and acts by binding pathogens and facilitating the entrapment in mucous and removal from the intestinal track [25]. Indeed a deficiency or inability to produce IgA results in frequent intestinal infections [26].

### Amino acids and the immune system

Although it has long been established that adequate nutrition is essential to the development and maintenance of the immune system, there is a rapidly growing body of literature that demonstrates the immune benefits of supplementation with specific nutrients, particularly during critical stages of development or disease states, when animals may have a higher demand for essential and non-essential nutrients. Such states include weaning, infectious diseases or chronic inflammatory conditions. The importance of individual amino acids to gut function and immunity has become apparent in recent years due to studies that have supplemented amino acids to animals/humans fed: 1) intravenously (total parenteral nutrition(TPN)), which demonstrates not only the importance of GALT but also the importance to immune functions beyond the intestine; 2) during weaning, which demonstrates the importance of these amino acids to the normal growth and development of the intestine and GALT; and 3) during infection or chronic inflammation, which has demonstrated the role in regulating inflammation and infectious challenges.

### Glutamine

Glutamine has been the most extensively studied amino acid with regards to its effects on GALT and the intestine. In health, glutamine is categorized as a non-essential amino acid and represents the amino acid in highest proportions in the body. However, during periods of stress and during critical stages of development the essentiality of exogenous sources of glutamine is now well-established to support growth [7] and health in young animals [27].

### Role as a precursor and energy substrate for immune and epithelial cells

Glutamine is an important energy substrate and precursor for other amino acids and derivatives in immune cells and enterocytes (Table 1) [2-4,28]. In fact, both cell types cannot function without at least some exogenous glutamine [29]. In immune cells, particularly lymphocytes, neutrophils and macrophages, glutamine is used rapidly and metabolized to glutamate, aspartate, lactate and CO_2_. Wu *et al.*[30] demonstrated the main metabolic fates of glutamine in enterocytes from weaning piglets are ammonia, glutamate, alanine, aspartate and CO_2_. As a precursor for glutamate, glutamine facilitates the production of glutathione (GSH), an important regulator of redox in enterocytes and lymphocytes [31]. It also provides nitrogen for the synthesis of nucleic acids and proteins that are needed for lymphocytes to proliferate and produce signals such as cytokines [32].

**Table 1:** Summary of the role of amino acids in GALT and the intestine

**Amino acid**	**Functions**
**Glutamine**	• Oxidative substrate for immune cells and IECs
	• Precursor for glutamate/GSH
	• Intestinal growth, structure and function (young animals and disease states)
	• Supports proliferative rates and reduces apoptosis of IECs
	• Protects against E.coli/LPS-induced damage to intestinal structure and barrier function
	• Lowers inflammatory and increases immunoregulatory cytokine production
	• Improves the proliferative responses of IELs and MLN cells
	• Intestinal IgA levels
	• Increases lymphocyte numbers in PP, lamina propria and IELs
**Glutamate**	• Oxidative substrate for immune cells and IECs
	• Precursor for GSH and other amino acids (i.e. arginine)
	• Intestinal growth, structure and function
	• Acts as Immunotransmitter between dendritic cells and T-cells*
	• Facilitates T-cell proliferation and Th1 and proinflammatory cytokine production
**Arginine**	• Precursor for NO and glutamate in IECs and immune cells
	• Intestinal growth, structure and function
	• Supports microvasculature of intestinal mucosa
	• Increases expression of HSP70 to protect intestinal mucosa
	• Protects against E.coli/LPS-induced damage to intestinal structure and barrier function
	• Facilitates neutrophil and macrophage killing through iNOS-mediated NO production
	• Increases intestinal IgA levels
	• Lowers inflammatory cytokine levels in intestine
	• Increases T-lymphocytes in lamina propria, PPs, intraepithelial spaces
**Methionine &Cysteine**	• Precursor for GSH, taurine and cysteine
	• Reduces intestinal oxidative stress
	• Intestinal structure
	• Increases goblet cells and proliferating crypt cells
	• Protects against DSS-induced intestinal damage (colitis model) by lowering inflammation, crypt damage and intestinal permeability.
**Threonine**	• Mucin synthesis
	• Intestinal structure and function
	• Intestinal IgA levels

*No direct evidence of effects on immune cells in GALT.

### Effects on intestinal function

In addition to its role as an energy substrate, glutamine is important for intestinal development and function, including maintaining the integrity of the gut barrier, the structure of the intestinal mucosa and redox homeostasis (Table 1).

Experimental evidence suggests that glutamine supplementation to weaning animals is beneficial to intestinal health. Wu *et al.*[28] first demonstrated that oral supplementation with 1% w/w glutamine prevented the decrease in jejunal villus height that occurs following weaning. Oral supplementation of glutamine (0.5-1.0% w/w) to healthy weaning piglets improves measures of intestinal health, including increasing villus height and crypt depth, reducing oxidative stress, lowering the proportion of apoptotic IECs and increasing the proliferative rates of IECs [33,34]. Glutamine supplementation has been demonstrated to reduce the adverse effects of TPN on intestinal function in healthy animals. A TPN solution containing 2% w/v glutamine has improved villus length, crypt depth, tight junction protein expression (occludin, JAM1 and ZO-1), and epithelial permeability [35-37].

In addition to the beneficial effects in healthy animals, we and others have demonstrated that glutamine supplementation may be protective to intestinal health during *E. coli* infection in animals at weaning (Table 1). We previously demonstrated that supplementing the weaning diet of piglets with glutamine (at 4.4% w/w) improved intestinal barrier function (decreased ion movement across mucosa), and maintained tight junction (claudin-1 and occludin) protein expression after an *E. coli* challenge [38]. Similarly, Yi *et al.*[39] reported that weaned piglets fed 2% w/w glutamine for 12 days prior to oral *E. coli* challenge maintained villus height, area and volume similar to uninfected piglets. Suckling piglets supplemented with oral glutamine (3.42 mmol/kg body weight) were protected against LPS-induced damage to the intestine [40]. Glutamine supplementation (5% w/w) was also reported to improve gut barrier function in a rat model of colitis [41].

### Effects on GALT

*In vitro* and *in vivo* studies have demonstrated the importance of glutamine to B- and T-lymphocyte, neutrophil and macrophage functions (as reviewed by [42]). *In vitro*, glutamine supports the proliferative response of T-cells, plasma cell generation, macrophage inflammatory cytokine production and phagocytosis of neutrophils and macrophages [42]. We and others have shown that glutamine supplementation lowers inflammatory cytokine levels, improves intestinal cytokine mRNA expression, increases immunoregulatory cytokine concentrations and increases the proliferative responses of MLN cells to a B- and T-cell mitogen (pokeweed mitogen) in healthy weaning piglets [22,34,38,43]. We also reported a lower proportion of IgA + cells in the MLN of weaning piglets fed glutamine relative to the control group, suggestive of less intestinal permeability and subsequently lower MLN lymphocyte activation with supplementation (Table 1) [22].

In addition to healthy weaning animals, there is support for a protective effect of glutamine in models of sepsis suggesting a therapeutic role for this amino acid in the infected animal (Table 1). Oral glutamine supplementation (1.1-2% w/v) prior to the induction of sepsis or endotoxemia increased the number of lymphocytes in PPs and lamina propria and normalized intestinal IgA levels of control animals [44-46]. Interestingly, even a single IV bolus of glutamine given immediately following the induction of sepsis appears to be protective. Previous studies have demonstrated that a 0.75 g/kg bolus of glutamine normalized systemic and intestinal inflammatory cytokine levels, increased the number of CD8αα + TCRαβ + and TCRγδ+/CD8αα + IELs , lowered the expression of inflammatory mediators in IELs and reduced IEL apoptosis [47,48]. Glutamine supplementation (4% w/w) also increased the proportion of IgA + cells in the lamina propria in rat models of short bowel syndrome [49].

The importance of glutamine to the intestine is also evident when provided systemically. In healthy mice, a TPN solution containing 2% w/v glutamine was reported to restore intestinal IgA levels, the total number of lymphocytes in PPs, IEL and lamina propria, and improved intestinal levels of regulatory cytokines, IL-10 and IL-4 [37,50,51].

### Summary

Overall, animal studies have demonstrated that dietary supplementation with glutamine (0.5%–5% w/w) is required to maintain a healthy intestinal mucosa and support several GALT functions during weaning (lymphocyte counts and proliferative responses, decreased inflammatory cytokine production and increased immunoregulatory cytokines), infection (increased lymphocytes and sIgA levels, decreased inflammatory cytokine levels and IEL apoptosis, intestinal barrier function and structure and IEL proliferation and decreased oxidative stress) and other intestinal inflammatory states (increased sIgA levels). Providing glutamine systemically (TPN studies) have established the importance of glutamine to the health of the intestinal barrier (maintained intestinal structure and function) and for some GALT and other mucosal immune responses (maintained sIgA, lymphocyte and regulatory cytokine levels).

### Glutamate

Glutamate is one of the most abundant dietary amino acids, but is found in very low concentrations in plasma [6,52]. This is likely the result of glutamate being a major energy substrate for intestinal epithelial cells [6]. It also serves as a precursor for other amino acids (L-alanine, L-aspartate, L-ornithine and L-proline) and for GSH in the intestine [53]. GSH is essential to maintaining the thiol redox state, which is vital to adequate functioning of enterocytes and immune cells (Table 1) [6].

### Effects on intestinal function

Glutamate has a very low capacity to cross biological membranes, and enterocytes contain glutamate transporters in the plasma membrane [54] making them one of the few cells that can rapidly transport and metabolize exogenous glutamate [55]. This contributes to glutamate’s recognition as the single most important oxidative substrate for IECs [55]. Dietary glutamate, as both a carbon and nitrogen donor, is the precursor of the conditionally essential amino acid, arginine [55]. Maintaining endogenous arginine synthesis in piglet enterocytes has been demonstrated to be essential for optimal growth [31].

*In vitro* and *in vivo* studies have reported that providing glutamate can modulate the intestinal epithelium (Table 1). In an *in vitro* model of intestinal hyperpermeability (Caco2 cells), glutamate treatment reduced hyperpermeabilty up to 30% [56]. Wu *et al.*[57] reported that weanling piglets fed 1% w/w dietary glutamate for 20 days had increased jejunal villus height, mucosal thickness and intestinal epithelial cell proliferation. Although the immune functions of the intestine were not specifically measured in these studies, these changes would be consistent with improved intestinal immune function. However, Tsuchioka *et al.*[58] reported that rats given TPN supplemented with glutamate (6.3% w/v) for 5 days had lower mucosal thickness and villous height in the small intestine relative to control TPN, suggesting a negative effect on the intestinal epithelium when glutamate is provided systemically.

### Effects on immune function and GALT

Although immune cells produce considerable amounts of glutamate when provided glutamine [4], investigations into the effects of glutamate on immune cells are limited. It has been recently reported that T-cells, B-cells, dendritic cells and macrophages express glutamate receptors [59,60], suggesting that glutamate likely has an important role in immune cell function. In support, Sturgill *et al.*[60] reported that purified B-cells and peripheral mononuclear cells produced more IgG and IgE when cultured with glutamate *in vitro*. In T-cells, glutamate may function as an immunotransmitter, akin to its role as a neurotransmitter, as extracellular concentrations of glutamate have been shown to regulate T-cell responses (Table 1). Pacheco *et al.*[61] demonstrated that dendritic cells release glutamate during antigen presentation to T-cells and this released glutamate influences T-cell proliferation and cytokine production. During the early stages of dendritic cell-T-cell interaction, glutamate binds to the constitutively expressed mGlu5R on T-cells to inhibit proliferation and cytokine production; however, later in the interaction glutamate binds to mGlu1R to induce T-cell proliferation and Th1 and proinflammatory cytokine production [61]. This study demonstrates that glutamate plays an essential role in regulating antigen-specific T-cell activation and suggests that the high concentrations of glutamate in the intestine may play an important role in T-cell regulation in the gut.

Despite glutamate being present in high concentrations in the intestinal lumen and immune cells having unique glutamate receptors, there have not been dietary studies that have directly assessed the effect on GALT. Due to the high oxidation rate of glutamate by enterocytes and immune cells, and its role as a precursor for GSH and other amino acids [62] it is reasonable to postulate that changes in the availability of glutamate modulates aspects of GALT (Table 1). We recently reviewed the evidence and presented a hypothesis for a novel role of glutamate receptors on immune cells as the means by which changes in glutamate availability modulates specific immune functions [6]. In that review, we proposed that due to its immunosuppressive effects at concentrations above plasma levels, glutamate may have a key role in the development and maintenance of oral tolerance [6], a unique aspect of immunity in the intestine.

Despite the lack of investigation into the immune modulating properties of glutamate on GALT, it is likely that it has an essential role. To date, the effects of glutamate on GALT have not been examined *in vivo*. However, it is likely that glutamate has an essential role as an oxidative substrate to both enterocytes and immune cells. It is also a precursor for the synthesis of GSH, which is required to protect the intestinal mucosa and optimize immune cell function. And, finally, glutamate is a precursor for arginine, the substrate for the synthesis of NO. A high rate of NO synthesis by neutrophils is required during the innate immune response to infection. This is an important role of the immune system in the intestine.

### Summary

Dietary glutamate appears essential for intestinal barrier function and likely other immune functions of the IEC, primarily as a precursor for GSH and as an oxidative substrate for enterocytes. Based on the available data, we can only hypothesize that the availability of glutamate to the cells in GALT has an immunoregulatory role. Studies conducted in systemic immune cells suggest that glutamate is essential for T-cell activation and B-cell immunoglobulin production and we postulate from indirect evidence that glutamate has a role in the induction of oral tolerance (that originates in GALT) and protection from enteric infections.

### Arginine

In most adult mammals, arginine is considered a dietary non-essential amino acid as it can be synthesized from glutamine, glutamate and proline, but becomes conditionally essential during periods of stress [63,64]. Moreover, the absence of arginine in the diet has been shown to have adverse effects in adults, including reproductive, metabolic and neurological derangements [29]. Arginine is classified as an essential amino acid in young mammals as endogenous synthesis cannot meet demands [29]. Several studies have demonstrated that arginine supplementation, either to the piglet’s diet or to the lactating sow, improves growth performance in piglets [65-68]. The immune system is particularly sensitive to changes in arginine availability during early development and various disease states.

### Metabolism

Arginine is the most plentiful nitrogen carrier in animals and is a precursor for urea, polyamines, proline, creatinine, agmatine, glutamate and protein [64]. Perhaps most importantly, for the immune system, arginine is the only precursor for nitric oxide synthase (all isoforms) for the synthesis of nitric oxide (NO). In both the intestine and immune system, NO is essential for optimal functioning, including regulating the inflammatory response, facilitating killing of microbes by neutrophils and macrophages, and facilitating lymphocyte functions [63].

### Effects on intestinal function

The structure and function of the intestine is sensitive to the amount of arginine in the diet during critical periods of development and disease states (Table 1). Studies have shown that arginine supplementation supports the growth and the development of the intestine and mucosal barrier in weanling piglets [65,69,70]. Dietary L-arginine supplementation ranging from 0.6% to 1.0% w/w increased intestinal growth, mucosa microvasculature (0.7% but not 1.2% w/w), villus height, crypt depth, and goblet cell counts in the piglets [65,69,70]. A proposed mechanism is that feeding arginine (0.6% w/w) increases expression of heat shock protein 70 (HSP70) which prevents protein denaturation and associated cellular stress [65].

In addition to supporting normal growth and development, supplementation with arginine has also been reported to reduce intestinal damage induced by *E. coli* derived LPS (Table 1). Sukhotnik *et al.*[71] demonstrated that arginine (2% w/v in drinking water) ameliorated the adverse effects of LPS on the rat intestine, including improving intestinal weight, villous height, epithelial cell proliferation and mucosal DNA and protein. In addition, arginine (0.5 or 1.0% w/w) supplemented to weaned piglets abolished the villous atrophy and morphological changes induced by LPS infection [72]. Arginine supplementation (1% v/v in water) lowered serum concentrations of endotoxin suggestive of improved gut permeability in a rat model of acute pancreatitis [73]. In support of this finding, other researchers have reported that arginine supplementation reduces bacterial counts in mesenteric lymph nodes (4% w/w arginine) [74] and improves gut barrier function (0.33 g/d arginine) [75].

### Effects on GALT

The immunomodulatory properties of L-arginine are well established and have been reviewed elsewhere [63,76,77]. Arginine has a fundamental role in both the innate and adaptive immune responses. One of the primary functions of arginine in leukocytes is as a substrate for inducible nitric oxide synthase (iNOS) to produce NO. Macrophages and neutrophils utilize NO to kill a variety of pathogens and malignant cells [63,76]. NO also appears to be important for B-cell development and T-cell receptor function [63]. The effects of arginine on GALT have been studied in both healthy and disease states and the available evidence suggest a beneficial effect on immune function.

Feeding arginine has been shown to be beneficial to GALT in inflammatory and trauma animal models, as well as healthy animals (Table 1). Rats fed diets containing 1% w/w arginine orally prior to the induction of acute pancreatitis had a higher proportion of T-helper cells and an increased ratio of CD4+:CD8+ cells in the intestinal lamina propria, as well as a greater concentration of fecal sIgA [73]. Similarly, Fan *et al.*[78] reported that supplementing arginine (1 g/kg) to severely burned mice for 7 days increased the number of lymphocytes isolated from PPs and intestinal IgA concentrations. Arginine supplemented mice (1 g/kg) also had intestinal cytokine profiles favouring a less inflammatory state (increased IL-4 and IL-10 and lower IFN-γ and IL-2) [78]. In chickens, feeding diets containing 2% w/w arginine improved intraepithelial cytotoxicity to viral infection and improved the antibody response to vaccine, suggesting effects on both cell types of the acquired immune system [79].

Animal models of TPN in both health and disease states have demonstrated that arginine supplementation can reverse the negative effects that TPN (not providing nutrients to the intestine) has on GALT. Mice supplemented with arginine (2 g/kg), prior to (oral diet) and following (TPN), had greater numbers of PPs and lymphocytes isolated from PPs, higher intestinal IgA levels and greater PHA-stimulated IL-10 production (splenocytes) relative to mice given no arginine prior to induction of sepsis [80]. This study suggests that dietary arginine may be essential to maintaining the intestinal immune system during acute infection. Despite these improvements in immune parameters, arginine supplementation in this model of sepsis did not significantly improve survival [80]. However, arginine supplementation to healthy animals fed by TPN also seems to have a similar beneficial effect on GALT. TPN supplemented with 1% w/v arginine given to healthy mice increased the proportion of αβTCR + T-cells and CD4+ T-cells in PPs and intraepithelial spaces compared to mice supplemented with 0.3% w/v arginine [81]. These studies strongly support an essential role for a systemic supply of arginine to maintaining GALT, particularly when the intestine is not receiving nutrients directly from the diet.

### Summary

There is considerable support that in health and stressed conditions oral ingestion of arginine (0.6% to 2% w/w) has a beneficial effect on GALT, with particular improvements in aspects of the acquired immune response. Arginine also supports the growth, development and maintenance of a healthy intestinal mucosa during critical periods of development (weaning) and under certain health conditions. These effects on the intestinal mucosa and GALT may be partly explained by arginine’s role as an essential precursor for NO.

### Other amino acids

#### Methionine and cysteine–sulfur containing amino acids

The dietary essentiality of methionine and conditional essentiality of cysteine to humans and animals has been well established [82,83]. Currently, there is little direct evidence demonstrating that these sulfur-containing amino acids alter immune function. However, indirectly their efficacy is supported by evidence that their metabolites (taurine, GSH and homocysteine) have immunomodulatory properties *in vitro*[82]. GSH (also see glutamate section) functions as a free radical scavenger and may support proper immune cell function through a role in T-cell proliferation, and inflammatory cytokine regulation [6,82,83]. GSH also has a crucial role in protecting the intestinal epithelium from electrophile and fatty acid hydroperoxide damage [29]. There is evidence that taurine and homocysteine have immunodulatory properties. Taurine is an end product of cysteine metabolism and diets devoid of taurine in cats resulted in reduced lymphocyte numbers, and mononuclear cells with impaired respiratory burst capacity [82]. *In vitro* evidence suggests that taurine chloramine can suppress NF-kappaB activation and pro-inflammatory cytokine (IL-6 and TNF-α) production and in stimulated macrophages [82]. In an *in vitro* model, homocysteine promoted monocyte activation and increased their adhesion to endothelial cells [84]. At present there are no feeding studies to provide direct support for the effect of homocysteine or taurine on immune function in GALT.

There is some evidence that dietary methionine and cysteine are important to ensure the health of the intestine and immune function during development and in inflammatory states (Table 1). For example, Bauchart-Thevret *et al.*[85] demonstrated that relative to healthy neonatal piglets fed a deficient diet, piglets supplemented with cysteine (0.25 g/kg) and methionine (25 g/kg) had less intestinal oxidative stress, improved villus height and area and crypt depth, higher number of goblet cells and Ki-67+ proliferative crypt cells. Cysteine also appears to be therapeutic in stressed inflammatory states, through improving intestinal inflammation and permeability. An infusion of L-cysteine (0.144 g/kg) given to pigs after DSS-induced colitis lowered mRNA expression of IL-8, MCP-1, MIP-1α, and MIP-2, and normalized IL-6, TNF- α, IFN-γ, IL-12, IL-1β and IL-10 in colon tissue [86]. In addition, less inflammatory cell infiltration, crypt damage and lower intestinal permeability were observed in the pigs supplemented with L-cysteine (Table 1) [86]. While these studies demonstrate the importance of sulfur containing amino acids to gut health in healthy and stressed animals, there is no direct evidence of the effects on lymphocyte or macrophage cell function in GALT.

### Threonine

Threonine is a dietary essential amino acid that has been shown to have a particularly high retention rate in the intestine, which suggests an important function in the gut [55,87]. Threonine has a major role in mucin synthesis, a glycoprotein that is required to protect the intestinal epithelium (Table 1) [88]. Mucin production is reduced in diets low or deficient in threonine in healthy rats and piglets [88-91]. Feeding a diet low in threonine (0.37% w/w) was found to adversely affect tight junction ultrastructure in the intestinal epithelium and induce villus atrophy in pigs [91,92], supporting the importance of a dietary supply of threonine in maintaining gut barrier function. Consistent with this, threonine-deficient piglets were found to have higher paracellular permeability which would increase the risk of infectious organisms or their products coming in contact with the body [92]. To date, there are no studies examining the effect of feeding threonine on the function of immune cells in GALT. However, Hamard *et al.*[92] reported that pigs fed a 30%-reduced threonine diet for two weeks had increased expression of genes involved in inflammation and immunity in the ileum, including MHC Class I antigen (HLA-B), T-cell differentiation antigen CD6, and chemokine receptors. Chickens fed 0.4% w/w threonine in the diet for 8 weeks had higher IgA concentrations in the ileum than chickens fed 0%, 0.1% or 0.2% threonine [93], suggesting an effect on B cell function in the lamina propria (Table 1).

## Conclusion

The intestine and the GALT are essential components of immune defense, protecting the animal/human from foreign antigens and pathogens, while allowing the absorption and tolerance of dietary nutrients. Feeding trials, primarily conducted in pigs and rodents, have established convincing evidence that not only the total protein intake but the availability of specific dietary amino acids, in particular glutamine, glutamate, and arginine, and perhaps methionine, cysteine and threonine, are essential to optimizing the immune functions of the intestine and specific immune cells located in GALT. These amino acids modulate their effects by maintaining the integrity, growth and immune functions of the epithelial cells in the intestine, as well as improve T-cell numbers and function, the secretion of IgA, and regulate inflammatory cytokine secretion. The studies conducted using feeding regimes (TPN) that bypass the oral route suggest that amino acids delivered in the blood from other parts of the body are important for maintaining GALT.

To date the majority of the studies have focussed on modulating single amino acids in a diet that contains many different proteins (combinations of amino acids) and determined function by measuring selective (often single parameters) functions. Evidence for some of these immunoactive amino acids comes primarily from *in vitro* studies or cells isolated from the systemic immune system (blood). Future studies should be designed using amino acid mixtures based on the existing knowledge to optimize immune function and growth in domestic animals and humans during critical periods of intestinal and GALT development in order to optimize health.

## Abbreviations

FAE: Follicle associated epithelium; HSP70: Heat shock protein 70; IEC: Intestinal epithelial cell; IEL: Intraepithelial lymphocyte; IgA: Immunoglobulin A; IL: Interleukin; iNOS: Inducible nitric oxide; GALT: Gut-associated lymphoid tissue; GSH: Glutathione; LPS: Lipopolysaccharide; MLN: Mesenteric lymph node; NO: Nitric oxide; PP: Peyer’s patches; sIgA: Secretory IgA; TCR: T-cell receptor; Th1: T-helper 1; TNF-α: Tumour necrosis factor-alpha; TPN: Total parenteral nutrition.

## Competing interests

MRR and CJF do not have any competing interests to disclose.

## Authors’ contributions

CJF conceived of the manuscript’s purpose and design and critically revised the manuscript. MRR wrote and revised the manuscript according to CJF’s suggestions. Both authors read and approved the final manuscript submitted.
